# Green Fabrication of Zinc Oxide Nanoparticles Using *Phlomis* Leaf Extract: Characterization and In Vitro Evaluation of Cytotoxicity and Antibacterial Properties

**DOI:** 10.3390/molecules26206140

**Published:** 2021-10-11

**Authors:** Amal A. Alyamani, Salim Albukhaty, Salman Aloufi, Faizah A. AlMalki, Hassan Al-Karagoly, Ghassan M. Sulaiman

**Affiliations:** 1 Department of Biotechnology, College of Science, Taif University, P.O. Box 11099, Taif 21944, Saudi Arabia; s.aloufi@tu.edu.sa; 2 Department of Chemistry, College of Science, University of Misan, Maysan 62001, Iraq; 3 Department of Biology, College of Science, Taif University, P.O. Box 11099, Taif 21944, Saudi Arabia; fa.ahmad@tu.edu.sa; 4 Department of Internal and Preventive Medicine, College of Veterinary Medicine, University of Al-Qadisiyah, Al-Diwaniyah 58002, Iraq; hassan.aliwee@qu.edu.iq; 5 Division of Biotechnology, Department of Applied Sciences, University of Technology, Baghdad 10066, Iraq; ghassan.m.sulaiman@uotechnology.edu.iq

**Keywords:** zinc dioxide nanoparticles, green synthesis, *Phlomis*, cytotoxicity, antibacterial activity

## Abstract

Green nanoparticle synthesis is an environmentally friendly approach that uses natural solvents. It is preferred over chemical and physical techniques due to the time and energy savings. This study aimed to synthesize zinc oxide nanoparticles (ZnO NPs) through a green method that used *Phlomis* leaf extract as an effective reducing agent. The synthesis and characterization of ZnO NPs were confirmed by UV-Vis spectrophotometry, Fourier Transform Infrared Spectroscopy (FTIR), X-ray Diffraction (XRD), Dynamic light scattering (DLS), Zeta potential, and Field Emission Scanning Electron Microscope (FESEM) techniques. In vitro cytotoxicity was determined in L929 normal fibroblast cells using MTT assay. The antibacterial activity of ZnO nanoparticles was investigated using a disk-diffusion method against *S. aureus* and *E. coli*, as well as minimum inhibitory concentration (MIC) and minimum bactericidal concentration (MBC) content concentrations. XRD results confirmed the nanoparticles’ crystalline structure. Nanoparticle sizes were found to be around 79 nm by FESEM, whereas the hydrodynamic radius of nanoparticles was estimated to be around 165 ± 3 nm by DLS. FTIR spectra revealed the formation of ZnO bonding and surfactant molecule adsorption on the surface of ZnO NPs. It is interesting to observe that aqueous extracts of *Phlomis* leave plant are efficient reducing agents for green synthesis of ZnO NPs in vitro, with no cytotoxic effect on L929 normal cells and a significant impact on the bacteria tested.

## 1. Introduction

Nanotechnology has made significant advances in the production of nanoparticles that may be used in various applications and the utilization of novel techniques and materials [1,2,3]. Traditional chemical and physical procedures may now produce large quantities of metal nanoparticles in a short time; however, these techniques result in the presence of some toxic chemicals adsorbed on the surface, which could have negative consequences in medical applications [4,5]. The development and discovery of novel non-toxic, environmentally friendly methods for the synthesis of metal nanoparticles, such as plants and bioactive elements found in plants, has garnered considerable attention due to their superior reducing ability and antimicrobial properties, as well as the physicochemical features of green NP synthesis; this technique also has the added benefit of increasing the life span of NPs, which overcomes the limits of traditional chemical and physical NP synthesis methods [6,7,8]. Metal oxide nanoparticles have generated considerable attention in biomedical technology due to their enormous surface area and are widely used in industrial and therapeutic applications [9,10,11]. Both metal and metal oxide nanoparticles exhibit substantial antioxidant and antibacterial properties and are frequently used in the detection of pathogenic microorganisms and in the diagnosis of cancer progression [12,13,14]. At low concentrations, metal oxide nanoparticles such as zinc (Zn), titanium (Ti), and magnesium (Mg) oxide nanoparticles limit microbial development [15,16,17]. Optical and structural properties of ZnO thin films produced by magnetron sputtering have been established in several studies [18], whereas several chemical techniques have been used for the synthesis of CdS nanoparticles using novel surfactants as stabilizing agents [19]. A recent study indicates that metal nanoparticles can be synthesized utilizing a green synthesis strategy that employs diverse plant components as a reducing agent [20]. The nanoparticles synthesized using this approach exhibited significant antioxidant and antibacterial activity while being less cytotoxic [21]. ZnO has recently attracted much interest among nanoparticles because of its unique qualities, such as its highly catalytic and photochemical activity [22]. Herbal medicines have long been known to be rich in pharmacologically active compounds. Plants have been utilized for various purposes, including medicine, flavour, dye, disinfectants, scents, cosmetics, charm, tobacco, and industrial applications. *Phlomis* is an annual herb plant that belongs to the Lamiaceae family and has over 100 species worldwide [23]. This genus of the plant is grown mainly in Europe, Africa, and Asia as well as Middle East countries like Saudi Arabia and Iraq. According to a review of several publications, *Phlomis* species contain aromatic compounds, polysaccharides, polyphenols, flavonoids, alkaloids, tannins, saponins, and terpenoids, which act as reductive agents and have medicinal and antibacterial activities [24,25,26]. *Phlomis* extracts can thus be used as reducing and capping agents, creating ZnO NPs by mixing with zinc salt solutions. To our knowledge, the synthesis of ZnO NPs using *Phlomis* and their antimicrobial and cytotoxicity properties are not well documented. Thus, the main objective of this study was to investigate the use of *Phlomis* leaves plant extract as a capping agent for the in vitro creation of ZnO NPs and test their cytotoxicity on normal fibroblast cell line (L929) using 3-[4,5-dimethylthiazol-2-yl]-2,5 diphenyl tetrazolium bromide (MTT) assay. The antibacterial activity against Gram-positive (*Staphylococcus aureus*) and Gram-negative bacteria (*Escherichia coli*) was performed using agar disc diffusion method, minimum inhibitory concentration (MIC), and minimum bactericidal concentration (MBC) content concentrations. Furthermore, this study might provide the basis for *Phlomis* to synthesize ZnO NPs, which are considered to be a low-cost, simple, and environmentally friendly approach that could be used for future biomedical applications.

## 2. Results and Discussion

In the green production of ZnO NPs, a *Phlomis* plant extract was employed. The color of the reaction mixture changed from pale yellow to dark brown after adding the plant leaf extract to the zinc nitrate solution, suggesting the synthesis of ZnO NPs. Green synthesis methodologies are biologically based on several parameters such as solvent, temperature, pressure, and pH conditions (acidic, basic, or neutral). Because of the availability of effective phytochemicals in various plant extracts, especially in leaves, such as ketones, aldehydes, flavones, amides, terpenoids, carboxylic acids, phenols, and ascorbic acids, functional groups in plant metabolites such as amine, hydroxyl, and carbonyl can interact with metal ions and decrease molecules to nanoscale size [27].

### 2.1. UV–Vis Absorption Techniques

**Figure 1:**
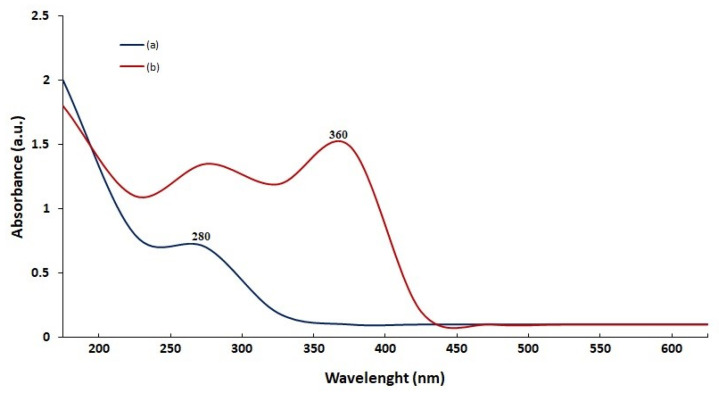
The UV-Vis absorption spectra of *Phlomis* leaf extract (**a**) and synthesized zinc oxide nanoparticles (**b**).

The optical properties of the produced ZnO NPs were evaluated using UV–Vis absorption techniques. In UV–Vis absorption spectra, electron transfers from the valence band to the conduction band assigned to the crucial bandgap energy of ZnO crystals result in a large absorption band at 360 nm as seen in Figure 1. UV–Vis absorption measurements for ZnO NPs were confirmed in most research in the 350–380 nm range. An absorption band around 280 nm was shown, which might be attributable to the electronic transitions of the various phenolic compounds in plant extract [28]. The spectra of the synthesized ZnO NPs displayed a peak at 360 nm due to intrinsic bandgap absorption, validating the ZnO NPs production. The bandgap energy was measured at 3.47 eV [29].

### 2.2. XRD Analysis

**Figure 2:**
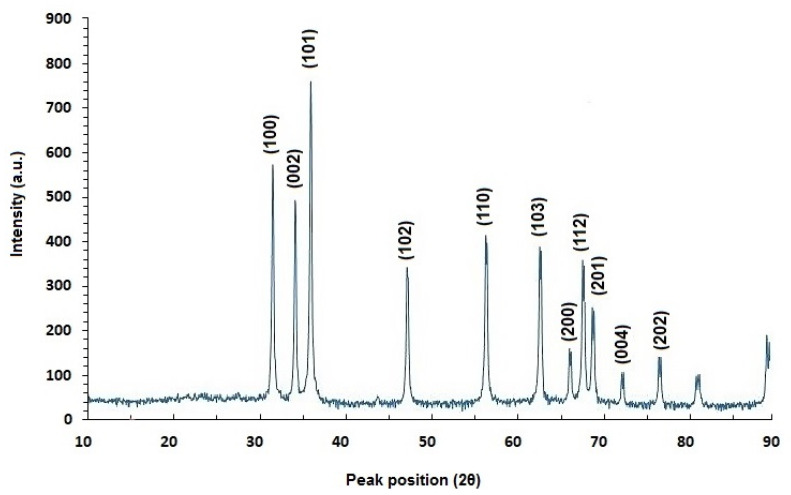
XRD patterns of green-synthesized ZnO NPs using *Phlomis* leaf extract. (Reference code: 96-900-4179).

Figure 2 showed the XRD pattern of green-synthesized ZnO NPs using *Phlomis* leaf extract. Diffraction peaks were shown at 2θ = 31.8341°, 34.4911°, 36.321°, 47.6034°, 56.6643°, 62.9192°, 66.4384°, 68.0045°, 69.1421°, 72.6285°, and 77.0281°, and were assigned to (100), (002), (101), (102), (110), (103), (200), (112), (201), (004), and (202) planes respectively.

According to established references, all of the diffraction peaks were properly indexed to the hexagonal phase of ZnO NPs.

### 2.3. DLS and Zeta Analyses

**Figure 3:**
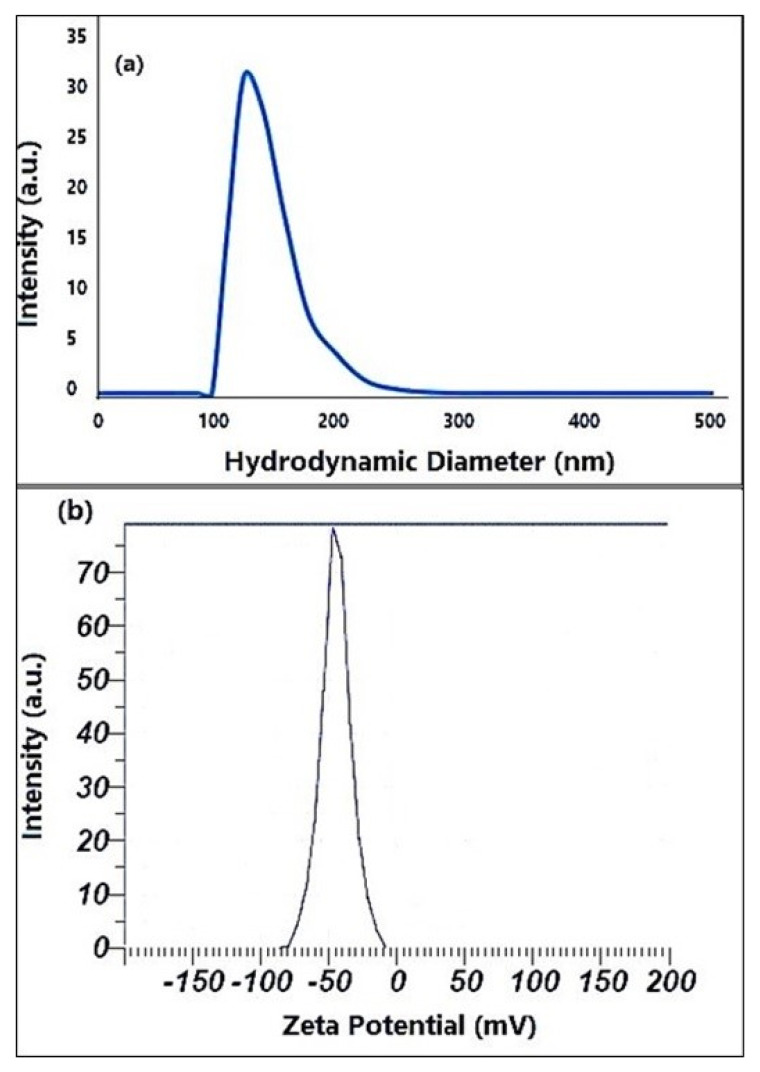
(**a**) The size distribution of prepared ZnO NPS by dynamic light scattering DLS. (**b**) Zeta potential value of prepared ZnO NPS.

Figure 3a,b demonstrates the DLS analysis experimental values and the zeta potential of produced ZnO nanoparticles, respectively. DLS analysis was used to measure the hydrodynamic size of the produced ZnO nanoparticles solution in this study, and the results are shown in Figure 3a. The investigation reveals a minor aggregation with a hydrodynamic diameter of 165 ± 3.0 nm on average. Figure 3b showed that the zeta potential of green-synthesized ZnO NPs with an adverse value of about 44 mV indicated a strong negative charge. Furthermore, the prepared suspension investigation reveals that the overall zeta potential criteria for improved stability are negative. The negative surface zeta potential of ZnO NPs formed following reduction could be due to surface-capped plant polyphenols that are adsorbed on ZnO NPs.

### 2.4. FESEM Assay

**Figure 4:**
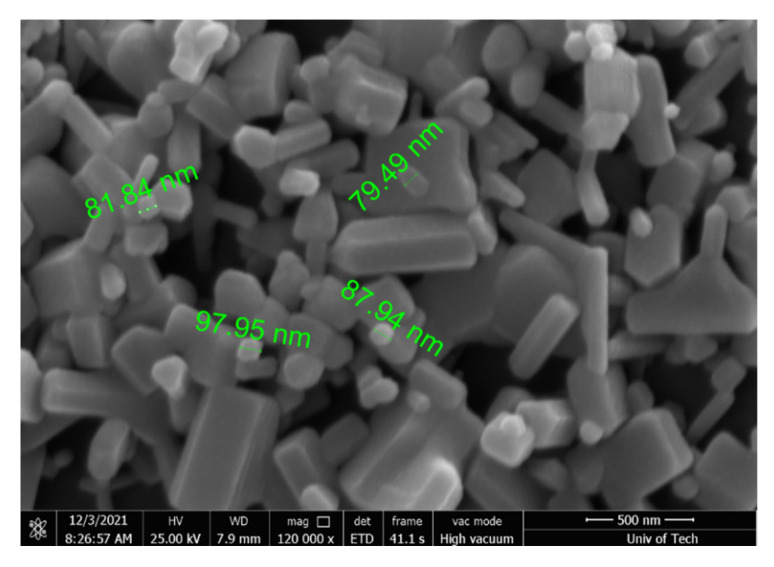
Field Emission scanning electron microscope (FESEM) image of green-synthesized ZnO NPs.

The size and shape of the compounds were seen using a FESEM technique. The synthesized ZnO nanoparticles’ SEM characterization data are displayed in Figure 4. The hexagonal form nanoparticles with an average particle size of 79 nm were visible in the FESEM image.

### 2.5. FTIR Results

**Figure 5:**
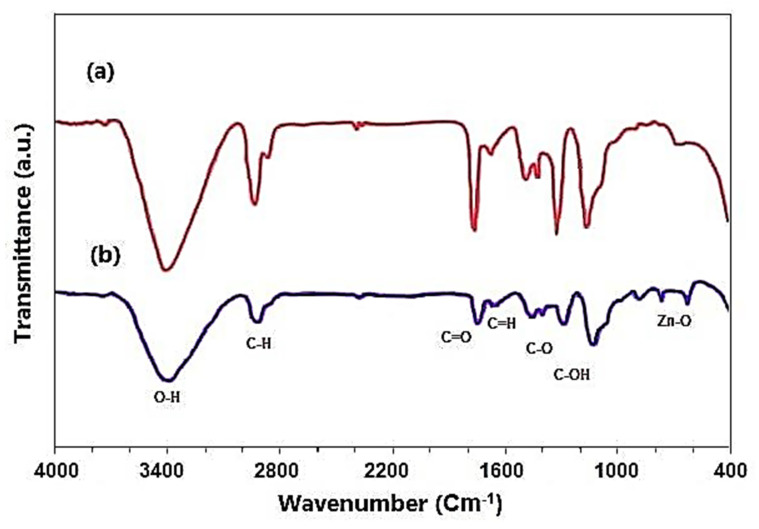
FTIR spectrum of *Phlomis* leaf extract (**a**) and (**b**) synthesized ZnO NPs.

FTIR analysis was used to determine the functional groups of *Phlomis* leaf extract and their role in the synthesis of ZnO NPs (Figure 5). FTIR showed a broad peak roughly at 3420 and 2921 cm^−1^ due to O-H stretching of alcohols and phenols. Besides, the absorption peak at 1719 cm^−1^ is related to C=C stretch due to the aromatic ring structure (aromatic). The vibrations of stretching are observed at 1225 cm^−1^, which represents C-O stretching. FTIR spectra of green-synthesized ZnO NPs revealed absorption bands at 601 and 496 cm^−1^, which is a characteristic of the Zn-O connection, which was identified, indicating that the substance is zinc oxide; these findings were in agreement with a study conducted by Senthilkumar et al. [29].

Flavonoids, iridoids, diterpenoids, phenylpropanoids, triterpenoids, and other phenolic compounds detected in *Phlomis* leaf extract may have played a role in nanoparticle formation, according to prior research [30]. Furthermore, as indicated by the foregoing results, the functional groups -OH (hydroxyl), -C=O (Carbonyl), and C-N (amine) found in the leaf extract were definitely implicated in the formation of ZnO NPs [31].

The biomolecules in the plant extract serve as effective capping agents, so helping in the synthesis of NPs. The capping agents appear to stabilize NPs by a variety of mechanisms, including electrostatic stability, steric stabilization, hydration force stabilization, and van der Waals forces. The stability of nanoparticles is significant for their functions and applications [32].

### 2.6. MTT Results

Conventional methods for the synthesis of ZnO NPs include microwave decomposition [33], simple wet chemistry routes [34], deposition processes, simple precipitation methods [35], hydrothermal synthesis [36], solvothermal methods [37], microwave hydrothermal methods [38], and hydrothermal techniques [39]. However, these physiochemical methods are expensive, time and energy-consuming, and generate multiple hazardous chemicals by-products. Thus, there is a need for a “green chemistry” approach to NP synthesis that includes clean, non-toxic, and environmentally friendly methods that can be applied in the ambient atmosphere. NPs synthesized via green synthetic routes are highly water-soluble, biocompatible, and less toxic. Plant extracts are a very promising tool for the facile green synthesis of NPs. Laurus nobilis leaf extract, Ziziphus jujuba leaf extract, and aloe vera leaf extracts were used in the synthesis of ZnO NPs [40,41,42].

**Figure 6:**
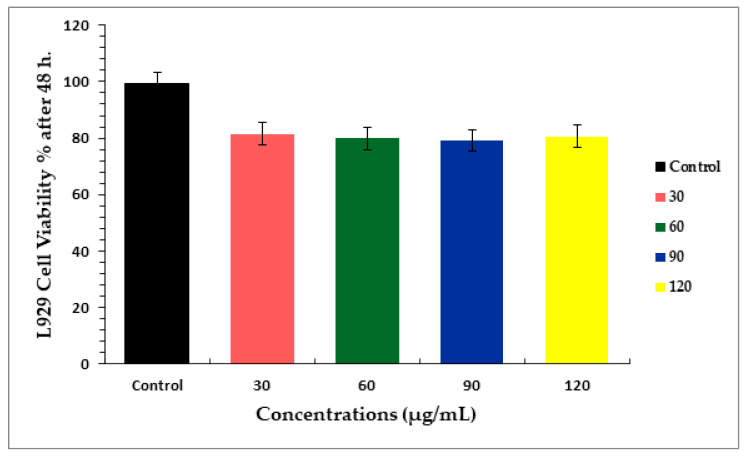
The MTT assay results of green-synthesized ZnO NPs on L929 normal fibroblast cell culture after 48 h. Cells were treated in a dose-dependent approach. The viability of the cells was evaluated using the MTT test, as detailed in the materials and methods section. Data displayed are mean ± standard deviation of three identical experimental tests done in triplicate.

The MTT assay was used for detection of the cytotoxicity of various doses of concentrations, 30, 60, 90, and 120 μg mL^−1^ of ZnO NPs for 48 h on L929 normal fibroblast cells. Figure 6 showed the percentage of cell viability in respective concentrations of ZnO NPs. Compared to control, the viability of cells of studied samples showed less cytotoxic effects (~20% of toxicity) at concentrations of 30, 60, 90, and 120 μg mL^−1^ for 48 h.

### 2.7. Antibacterial Properties

**Figure 7:**
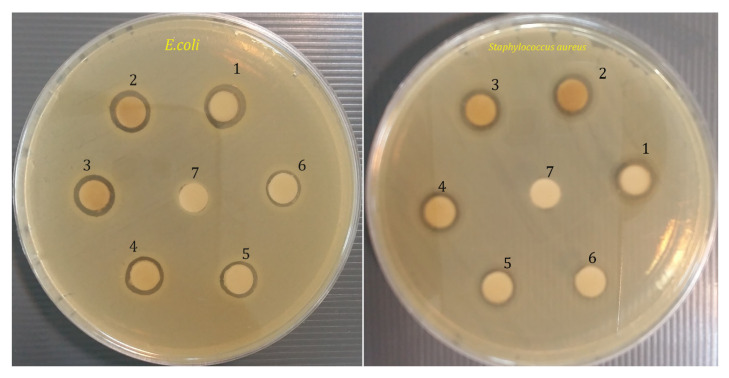
Zone of inhibition produced by green-synthesized ZnO-NPS against bacterial strains: *E. coli and S. aureus*. 1: Chloramphenicol (positive control), 2: 2000 (µg/mL), 3: 1000 (µg/mL), 4: 500 (µg/mL), 5: 250 (µg/mL), 6: 125 (µg/mL), 7: Distillated water (Negative control).

The antibacterial activity of green-ZnO NPs against *S. aureus* and *E. coli* are shown in Table 1, Table 2 and Figure 7. The findings revealed that ZnO NPs exhibit antibacterial activity against the target bacteria at varying doses. The most significant spectrum of activity was seen in ZnO NPs. *S. aureus* had the highest MIC of 250 µg mL^−1^ and *E. coli* had the lowest MIC of 125 µg mL^−1^, and zinc oxide nanoparticles had the most antibacterial action. The effect of their MIC on the microorganisms tested was significantly different (*p* < 0.05).

The ZnO NPs synthesized at suitable laboratory conditions were used to check the antibiotic sensitivity at five different concentrations (125, 250, 500, 1000, and 2000 µg mL^−1^) as shown in Table 1, which exhibited ZnO’s inhibition zone NPs against tested bacteria. The maximum zone of inhibition was around 16.8 ± 0.1 mm for *E. coli* and 15.1 ± 0.2 for *S. aureus* at 2000 µg mL^−1^ of ZnO NPs concentration. On the other hand, the negative control (distilled water) did not exhibit any zone of inhibition. The positive control (chloramphenicol) displayed antimicrobial activity against both tested bacteria, *S. aureus* and *E. coli*, (27.0 ± 0.2 and 27.8 ± 0.2 mm), respectively. The mechanism action of ZnO NPs as an antibacterial agent against many bacteria is yet unknown and needs to be investigated extensively [43]. The effect of nanoparticle size on bacteria may be due to direct or electrostatic contact of tiny ZnO NPs with the cell membrane, cell internalization of ZnO NPs, and the generation of active oxygen species. Direct interaction of ZnO NPs with the bacterial cell surface alters the permeability of the cell membrane toward the NPs, according to these hypotheses [44].

Recent studies have demonstrated that ZnO NPs damage bacterial cell membranes, causing intracellular component lysis and, finally, bacterial cell death [45].

ZnO NPs may have adhered to the cell surface membrane of bacteria, resulting in disrupting processes such as permeability and respiration. As a result, the ability of particles to bind to bacteria is clearly dependent on the amount of surface area obtained for interaction. Commonly, small nanoparticles have a higher surface area for bacterial invasion than bigger particles due to their stronger antibacterial activity [46]. According to our findings, Gram-positive bacteria had a smaller inhibitory zone than Gram-negative bacteria. This could be because Gram-positive bacteria have thicker, more solid multiple layers of peptidoglycan in their cell walls, which inhibits nanoparticles from penetrating [47]. In recent studies, ZnO NPs produced from *Butea monsoperma*, *Acacia nilotica* (L.), and *Plectranthus amboinicus* leaf extracts demonstrated high antibacterial activity against *Pseudomonas aeruginosa*, *Klebsiella Pneumoniae*, and *Staphylococcus aureus* [48,49,50], suggesting that traditional medicinal extract-mediated ZnO NP synthesis could be extremely beneficial for the medical industries.

This study was carried out to further strengthen the effect of antibacterial activity concerning the physicochemical characteristics of ZnO NPs synthesized by a green method using *Phlomis* leaf extract as a reducing factor. Due to their superior reduction capacity and antibacterial activity, using plant and plant-based bioactive chemicals to create metal nanoparticles is becoming critical. This technique is known as green synthesis [51]. The size and shape of the nanoparticles are determined by the type of plant and plant extract used. The synergistic interaction of bioactive chemicals found in plants and nanomaterial precursors employed for synthesis is responsible for the increased biological activity of green-produced nanoparticles. Due to the improved electrochemical performance of biosynthesized nanoparticles, green produced nanoparticles have also exhibited various features such as electrochemical detection of numerous antibiotic drugs. The enhanced bioactivity of smaller particles probably is attributed to the higher surface area to volume ratio [52].

## 3. Materials and Methods

### 3.1. Leaves Extract Preparation

*Phlomis* was collected from Taif governorate, KSA, and botanists Taif University confirmed the authenticity. The extraction of *Phlomis* Leaves was carried out in accordance with the previously described study with some modification [53]. Fresh leaves of *Phlomis* were collected and cleaned with tap water before being washed with deionized water; the leaf sample was allowed to dry. The dried leaves were crushed and powdered in a sterilized electric blender, then 35 g of the dried leaves powder was weighed and heated for 2 h at 90 °C with 100 mL deionized water under magnetic stirring. After cooling to room temperature, the extract solution was filtered through sterile Whatman No. 1 filter paper and stored at 4 °C until further assessment.

### 3.2. Green Synthesis of ZnO NPs

The green synthesis of ZnO NPs was carried out in accordance with a previously described study [54] with some modifications. Briefly, 30 mL of *Phlomis* leaf aqueous extract was boiled using a Magnetic stirrer with a hot plate at 85 °C. When the solution reached 65 °C, 3 g Zinc Nitrate Hexahydrate (Zn(NO_3_)_2_·6H_2_O; Sigma-Aldrich, Saint-Louis, MO, USA) was added and left to boil until the extract became a paste. It was then placed in a ceramic crucible cup and cooked for 3 h in a furnace at 350 °C. The powder of synthesized ZnO NPs was collected, then re-suspended in deionized distilled water, and centrifuged for another time. This step was repeated twice to ensure removal of the residual zinc nitrate and plant filtrate. After that, the powdered material (ZnO NPs) was used in the subsequent research.

### 3.3. Characterisation of ZnO NPs

UV–Vis spectroscopy (UV-1800 UV-Vis Spectrophotometer from (Shimadzu, Tokyo, Japan) in the 200–800 nm range was utilized to characterize nanoparticles to confirm the produced green-synthesized ZnO NPs. To determine the functional groups of the specimens, Fourier Transform Infrared Spectroscopy (FTIR) was used to analyze them over a wavelength range of 400–4000 cm^−1^. The crystalline structure form of the produced nanoparticles was analyzed using a Bruker D8 Advance diffractometer (Billerica, MA, USA) with CuKα *radiation* (λ *=* 1.5418 Å). Furthermore, the morphology and size of green-synthesized ZnO NPs were examined by field-emission scanning electron microscopy (FESEM, HITACHI, S-4160, Tokyo, Japan). Thin films of the ZnO NPs were prepared on a cover slide grid by dropping a little amount of sample over the cover slide grid and allowing it to dry at room temperature before visualizing under FESEM.

DLS and Zeta analyses of ZnO NPs were performed using a particle size analyzer (Malvern Zetasizer, Malvern, England) and zeta potential measurement using (Horiba SZ-100 nanoparticle analyzer) to investigate particles’ size and surface charge of prepared NPs. The electrostatic potential of the particles was determined using an ultrasonic dispersion of 0.01 g 100 mL^−1^ in DMSO at room temperature.

### 3.4. MTT Assay

MTT was employed according to the manufacturer’s instructions to determine the cytotoxic effect and biocompatibility of produced ZnO NPs against normal fibroblast cell line (L929). At 37 °C in 5% CO_2_, cells were cultivated in 96-well plates at a concentration of 1 × 10^5^ cells per well. After 24 h, ZnO NPs were added at four different concentrations (30, 60, 90, and 120 g mL^−1^), and the cells were washed twice with phosphate buffer saline PBS before each well was refilled with new 100 µL of culture media and 0.5 mg mL^−1^ of MTT reagent. The cells that were not labeled served as the control group. Following that, the labeled cells were cultured for four hours at 37 °C in 5% CO_2_. After 4 h, the medium was gently aspirated and reconstituted with new DMSO at a concentration of 100 g mL^−1^. The optical density (OD) at 570 nm with a reference filter of 650 nm was used to measure the amount of decreased MTT using a microplate reader SYNERGY-H1 (Biotek, Winooski, VT, USA). The following equation was used to determine the IC_50_ value:

Cell viability = Absorbance of sample/Absorbance of control × 100

### 3.5. Antibacterial Activity

The antibacterial activity of ZnO NPs synthesized from *Phlomis* plant extract was tested against Gram-positive and Gram-negative bacteria including *Staphylococcus aureus* (ATCC 29213) and *Escherichia coli* (ATCC 35218), using the disk diffusion method.

Briefly, Mueller–Hinton agar plates (Merck, Darmstadt, Germany) were sterilized and allowed to solidify. After solidification, a sterile glass rod was used to inoculate 30 µL of each bacterial suspension onto the Petri plates. Bacteria were inoculation by streaking the swabs on Muller–Hinton agar plates. To establish the interaction of these nanoparticles particles with the bacteria, a typical antibiotic disc was impregnated with 30 µL of zinc oxide suspension at concentrations of 125, 250, 500, 1000, and 2000 µg mL^−1^, and placed on Muller–Hinton agar plates. Distilled water and chloramphenicol were used as negative and positive control, respectively. To determine antibacterial activity, plates were incubated at 37 °C for 24 h. This activity (inhibitory zone) was quantified in millimeters. Triplicate tests were performed.

#### MIC and MBC Assays

The minimum inhibitory concentration (MIC) of an antibacterial agent is the lowest concentration at which bacterial growth is inhibited. The MIC of biosynthesized ZnO NPs against bacteria was determined using 2,3,5-triphenyl tetrazolium chloride (TTC) (Merck, Darmstadt, Germany) in a 96-well microtiter plate, according to CLSI guidelines [30]. With minor modifications, overall, the bacterial culture was grown to hold out a 0.5 Mc-Farland grade. Thereafter, 10 µL of bacterial suspension were pipetted into 140 µL of nutritional broth with varied concentrations of ZnO NPs (125–2000 µg mL^−1^). Nutrient broth without ZnO NPs was used as a control. The microtiter plate was incubated for 24 h at 37 °C. After that, each well was filled with approximately 10 µL of TTC solution at a concentration of 10 mg/mL and incubated at 37 °C for 180 min. The MIC value was properly considered in the wells that did not produce a red color.

Furthermore, the MBC is the lowest antimicrobial concentration that completely kills the bacteria and prevents bacterial growth. The MBC was determined by aseptically subculturing the well suspension from the MIC findings on nutritional agar. Approximately 10 µL of bacterial suspension was put onto an agar plate and incubated at 37 °C for 24 h. The MBC was considered as the lowest concentration that did not show any bacterial growth. All of the experiments were done in triplicate.

## 4. Conclusions

In this work, we used *Phlomis* leaf extract to synthesis ZnO NPs in a green and environmentally-friendly method. The shift in color of the reaction solution from brownish-yellow to pale white is the first sign of ZnO NPs. The NPs synthesized were 79 nm in size, according to SEM analysis. According to the XRD examination, the ZnO NPs generated had a highly pure and crystalline nature. The antibacterial activities of the produced ZnO NPs were impressive. *Phlomis* was discovered to have the potential to be exploited in the production of metal nanoparticles on a large scale.

## Figures and Tables

**Table 1:** Zone of inhibition (mm) of blank disks, ZnO against tested bacteria.

Compounds (µg mL^−1^)	Zone of Inhibition (mm) Average ± Standard Deviation	
	*S. aureus*	*E. coli*
* Distillated water	6.3 ± 0.2	6.3 ± 0.2
125	7.0 ± 0.2	8.0 ± 0.2
250	7.3 ± 0.1	8.2 ± 0.1
500	8.0 ± 0.1	8.4 ± 0.2
1000	9.1 ± 0.2	9.8 ± 0.1
2000	9.2 ± 0.2	9.8 ± 0.1
* Chloramphenicol	10.2 ± 0.1	10.7 ± 0.2

* Distilled sterile water and Chloramphenicol (10 µg) were used as positive and negative controls.

**Table 2:** Measured of MIC and MBC for ZnO NPS.

Bacteria	ZnO NPs Effect	
	MIC μg mL	MBC μg mL
*E. coli*	125	250
*S. aureus*	250	500

## Data Availability

Not applicable.

## Sample Availability

Samples of the compounds are not available from authors.

## References

[1] Goesmann H., Feldmann C. (2010). Nanoparticulate functional materials. Angew. Chem. Int. Ed..

[2] Albukhaty S., Naderi-Manesh H., Tiraihi T., Sakhi Jabir M. (2018). Poly-l-lysine-coated superparamagnetic nanoparticles: A novel method for the transfection of pro-BDNF into neural stem cells. Artif. Cells Nanomed. Biotechnol..

[3] Jabir M., Sahib U.I., Taqi Z., Taha A., Sulaiman G., Albukhaty S., Al-Shammari A., Alwahibi M., Soliman D., Dewir Y.H. (2020). Linalool-loaded glutathione-modified gold nanoparticles conjugated with CALNN peptide as apoptosis inducer and NF-κB translocation inhibitor in SKOV-3 cell line. Int. J. Nanomed..

[4] Al-Kaabi W.J., Albukhaty S., Al-Fartosy A.J.M., Al-Karagoly H.K., Al-Musawi S., Sulaiman G.M., Dewir Y.H., Alwahibi M.S., Soliman D.A. (2021). Development of *Inula graveolens* (L.) Plant Extract Electrospun/Polycaprolactone Nanofibers: A Novel Material for Biomedical Application. Appl. Sci..

[5] Ray P.C., Yu H., Fu P.P. (2009). Toxicity and environmental risks of nanomaterials: Challenges and future needs. J. Environ. Sci. Health Part C.

[6] Jihad M.A., Noori F., Jabir M.S., Albukhaty S., AlMalki F.A., Alyamani A.A. (2021). Polyethylene Glycol Functionalized Graphene Oxide Nanoparticles Loaded with Nigella sativa Extract: A Smart Antibacterial Therapeutic Drug Delivery System. Molecules.

[7] Safat S., Buazar F., Albukhaty S., Matroodi S. (2021). Enhanced sunlight photocatalytic activity and biosafety of marine-driven synthesized cerium oxide nanoparticles. Sci. Rep..

[8] Al-Musawi S., Albukhaty S., Al-Karagoly H., Sulaiman G.M., Alwahibi M.S., Dewir Y.H., Soliman D.A., Rizwana H. (2020). Antibacterial activity of honey/chitosan nanofibers loaded with capsaicin and gold nanoparticles for wound dressing. Molecules.

[9] Hashem M., Al-Karagoly H. (2021). Synthesis, characterization, and cytotoxicity of titanium dioxide nanoparticles and in vitro study of its impact on lead concentrations in bovine blood and milk. J. Biotech. Res..

[10] Al-Rahim A.M., AlChalabi R., Al-Saffar A.Z., Sulaiman G.M., Albukhaty S., Belali T., Ahmed E.M., Khalil K.A. (2021). Folate-methotrexate loaded bovine serum albumin nanoparticles preparation: An in vitro drug targeting cytokines overwhelming expressed immune cells from rheumatoid arthritis patients. Anim. Biotechnol..

[11] Khashan K.S., Sulaiman G.M., Abdulameer F.A., Albukhaty S., Ibrahem M.A., Al-Muhimeed T., AlObaid A.A. (2021). Antibacterial Activity of TiO2 Nanoparticles Prepared by One-Step Laser Ablation in Liquid. Appl. Sci..

[12] Konopko A., Kusio J., Litwinienko G. (2020). Antioxidant Activity of Metal Nanoparticles Coated with Tocopherol-Like Residues—The Importance of Studies in Homo- and Heterogeneous Systems. Antioxidants.

[13] Gold K., Slay B., Knackstedt M., Gaharwar A.K. (2018). Antimicrobial Activity of Metal and Metal-Oxide Based Nanoparticles. Adv. Ther..

[14] Ibrahim A.A., Kareem M.M., Al-Noor T.H., Al-Muhimeed T., AlObaid A.A., Albukhaty S., Sulaiman G.M., Jabir M., Taqi Z.J., Sahib U.I. (2021). Pt (II)-Thiocarbohydrazone Complex as Cytotoxic Agent and Apoptosis Inducer in Caov-3 and HT-29 Cells through the P53 and Caspase-8 Pathways. Pharmaceuticals.

[15] Albukhaty S., Al-Karagoly H., Dragh M.A. (2020). Synthesis of zinc oxide nanoparticles and evaluated it’s activity against bacterial isolates. J. Biotechnol. Res..

[16] Wang N., Fuh J.Y.H., Dheen S.T., Senthil Kumar A. (2021). Functions and applications of metallic and metallic oxide nanoparticles in orthopedic implants and scaffolds. J. Biomed. Mater. Res. Part B Appl. Biomater..

[17] Parham S., Wicaksono D.H., Bagherbaigi S., Lee S.L., Nur H. (2016). Antimicrobial treatment of different metal oxide nanoparticles: A critical review. J. Chin. Chem. Soc..

[18] Gao W., Li Z. (2004). ZnO thin films produced by magnetron sputtering. Ceram. Int..

[19] De La C.T.E.C., Ambrosio L.R.C., Mota G.M.L., Luqueb P.A., Castillo S.J., Carrillo-Castillo A. (2015). A simple method for the synthesis of CdS nanoparticles using a Novel surfactant. Chalcogenide Lett..

[20] Shanker U., Jassal V., Rani M., Kaith B.S. (2016). Towards green synthesis of nanoparticles: From bio-assisted sources to benign solvents. A review. Int. J. Environ. Anal. Chem..

[21] Rivera-Rangel R.D., González-Muñoz M.P., Avila-Rodriguez M., Razo-Lazcano T.A., Solans C. (2018). Green synthesis of silver nanoparticles in oil-in-water microemulsion and nano-emulsion using geranium leaf aqueous extract as a reducing agent. Colloids Surf. A Physicochem. Eng. Asp..

[22] Lee K.M., Lai C.W., Ngai K.S., Juan J.C. (2016). Recent developments of zinc oxide based photocatalyst in water treatment technology: A review. Water Res..

[23] Uritu C.M., Mihai C.T., Stanciu G.D., Dodi G., Alexa-Stratulat T., Luca A., Leon-Constantin M.M., Stefanescu R., Bild V., Melnic S. (2018). Medicinal plants of the family Lamiaceae in pain therapy: A review. Pain Res. Manag..

[24] Zhang Y., Wang Z.H. (2009). Phenolic composition and antioxidant activities of two Phlomis species: A correlation study. CR Biol..

[25] Limem-Ben Amor I., Boubaker J., Ben Sgaier M., Skandrani I., Bhouri W., Neffati A., Kilani S., Bouhlel I., Ghedira K., Chekir-Ghedira L. (2009). Phytochemistry and biological activities of phlomis species. J. Ethnopharmacol..

[26] Karadağ A.E., Demirci B., Kültür Ş., Demirci F., Başer K.H.C. (2020). Antimicrobial, anticholinesterase evaluation and chemical characterization of essential oil Phlomis kurdica Rech. fil. Growing in Turkey. J. Essent. Oil Res..

[27] Marslin G., Siram K., Maqbool Q., Selvakesavan R.K., Kruszka D., Kachlicki P., Franklin G. (2018). Secondary Metabolites in the Green Synthesis of Metallic Nanoparticles. Materials.

[28] Aleixandre-Tudo J.L., Du Toit W., Solis-Oviedo R.L., De La Cruz Pech-Canul A. (2018). The role of UV-visible spectroscopy for phenolic compounds quantification in winemaking. Frontiers and New Trends in the Size of Fermented Food and Beverages.

[29] Senthilkumar N., Nandhakumar E., Priya P., Soni C., Vimalan M., Vetha N. (2017). Synthesis of ZnO nanoparticles using leaf extract of *Tectona grandis* (L.) and their anti-bacterial, anti-arthritic, anti-oxidant and in vitro cytotoxicity activities. New J. Chem..

[30] Anjum S., Abbasi B.H. (2016). Biomimetic synthesis of antimicrobial silver nanoparticles using in vitro-propagated plantlets of a medicinally important endangered species: Phlomis bracteosa. Int. J. Nanomed..

[31] Gonçalves R.A., Toledo R.P., Joshi N., Berengue O.M. (2021). Green Synthesis and Applications of ZnO and TiO_2_ Nanostructures. Molecules.

[32] Kalpana V.N., Rajeswari V.D. (2018). A review on green synthesis, biomedical applications, and toxicity studies of ZnO NPs. Bioinorg. Chem. Appl..

[33] Sherlya E.D., Vijaya J.J., Selvam N.C.S., Kennedy L.J. (2014). Microwave assisted combustion synthesis of coupled ZnO–ZrO2 nanoparticles and their role in the photocatalytic degradation of 2,4-dichlorophenol. Ceram. Int..

[34] Cao H.L., Qian X.F., Gong Q., Du W.M., Ma X.D., Zhu Z.K. (2006). Shape- and size-controlled synthesis of nanometre ZnO from a simple solution route at room temperature. Nanotechnology.

[35] Bagheri S., Chandrappa K., Hamid S.B.A. (2013). Facile synthesis of nano-sized ZnO by direct precipitation method. Pharm. Chem..

[36] Zhou H., Fan T., Zhang D. (2007). Hydrothermal synthesis of ZnO hollow spheres using spherobacterium as biotemplates. Microporous Mesoporous Mater..

[37] Bai X., Li J., Liu H., Tan L., Liu T., Meng X. (2015). Solvothermal synthesis of ZnO nanoparticles and anti-infection application in vivo. ACS Appl. Mater. Interfaces.

[38] Huang J., Xia C., Cao L., Zeng X. (2008). Facile microwave hydrothermal synthesis of zinc oxide one-dimensional nanostructure with three-dimensional morphology. Mater. Sci. Eng. B.

[39] Aneesh P.M., Vanoja M.A., Jayaraj M. (2007). Synthesis of ZnO nanoparticles by hydrothermal method. Nanophotonic Mater IV Int. Soc. Opt. Photonics.

[40] Vijayakumar S., Vaseeharan B., Malaikozhundan B., Shobiya M. (2016). Laurus nobilis leaf extract mediated green synthesis of ZnO nanoparticles: Characterization and biomedical applications. Biomed. Pharmacother..

[41] Alharthi M.N., Ismail I., Bellucci S., Khdary N.H., Abdel Salam M. (2021). Biosynthesis Microwave-Assisted of Zinc Oxide Nanoparticles with Ziziphus jujuba Leaves Extract: Characterization and Photocatalytic Application. Nanomaterials.

[42] Sangeetha G., Rajeshwari S., Venckatesh R. (2011). Green Synthesis of Zinc Oxide Nanoparticles by *Aloe Barbadensis* Miller Leaf Extract: Structure and Optical Properties. Mater. Res. Bull..

[43] Basnet P., Chanu T.I., Samanta D., Chatterjee S. (2018). A review on bio-synthesized zinc oxide nanoparticles using plant extracts as reductants and stabilizing agents. J. Photochem. Photobiol. B Biol..

[44] Sánchez-López E., Gomes D., Esteruelas G., Bonilla L., Lopez-Machado A.L., Galindo R., Cano A., Espina M., Ettcheto M., Camins A. (2020). Metal-Based Nanoparticles as Antimicrobial Agents: An Overview. Nanomaterials.

[45] Shaikh S., Nazam N., Rizvi S.M.D., Ahmad K., Baig M.H., Lee E.J., Choi I. (2019). Mechanistic Insights into the Antimicrobial Actions of Metallic Nanoparticles and Their Implications for Multidrug Resistance. Int. J. Mol. Sci..

[46] Abdal Dayem A., Hossain M.K., Lee S.B., Kim K., Saha S.K., Yang G.-M., Choi H.Y., Cho S.-G. (2017). The Role of Reactive Oxygen Species (ROS) in the Biological Activities of Metallic Nanoparticles. Int. J. Mol. Sci..

[47] Hoseinzadeh E., Alikhani M.-Y., Samarghandi M.-R., Shirzad-Siboni M. (2014). Antimicrobial potential of synthesized zinc oxide nanoparticles against gram positive and gram-negative bacteria. Desalination Water Treat..

[48] Ali S.G., Ansari M.A., Alzohairy M.A., Alomary M.N., Jalal M., AlYahya S., Asiri S.M.M., Khan H.M. (2020). Effect of Biosynthesized ZnO Nanoparticles on Multi-Drug Resistant *Pseudomonas aeruginosa*. Antibiotics.

[49] Rasha E., Monerah A., Manal A., Rehab A., Mohammed D., Doaa E. (2021). Biosynthesis of Zinc Oxide Nanoparticles from *Acacia nilotica* (L.) Extract to Overcome Carbapenem-Resistant *Klebsiella Pneumoniae*. Molecules.

[50] Vijayakumar S., Vinoj G., Malaikozhundan B., Shanthi S., Vaseeharan B. (2015). *Plectranthus amboinicus* leaf extract mediated synthesis of Zinc Oxide nanoparticles and its control of methicillin-resistant *Staphylococcus aureus* biofilm and blood sucking mosquito larvae. Spectrochim. Acta A.

[51] Singh A., Gautam P.K., Verma A., Singh V., Shivapriya P.M., Shivalkar S., Sahoo A.K., Samanta S.K. (2020). Green synthesis of metallic nanoparticles as effective alternatives to treat antibiotics resistant bacterial infections: A review. Biotechnol. Rep..

[52] Khan I., Saeed K., Khan I. (2019). Nanoparticles: Properties, applications and toxicities. Arab. J. Chem..

[53] Allafchian A.R., Mirahmadi-Zare S.Z., Jalali S.A., Hashemi S.S., Vahabi M.R. (2016). Green synthesis of silver nanoparticles using phlomis. J. Nanostruct. Chem..

[54] Rad S.S., Sani A.M., Mohseni S. (2019). Biosynthesis, characterization and antimicrobial activities of zinc oxide nanoparticles from leaf extract of *Mentha pulegium* (L.). Microb. Pathog..

